# Current Perspectives on the Determinants of Acceptability of Pre-Exposure Prophylaxis and Nonoccupational Post-Exposure Prophylaxis among People at Risk for HIV: A Scoping Review

**DOI:** 10.3390/ijerph191912605

**Published:** 2022-10-02

**Authors:** Lingmi Zhou, Sawitri Assanangkornchai

**Affiliations:** 1 Department of Epidemiology, Faculty of Medicine, Prince of Songkla University, Hat Yai, Songkhla 90110, Thailand; 2 Department of AIDS Control and Prevention, Guilin Center for Disease Control and Prevention, Guilin 541000, China

**Keywords:** acceptability, HIV, nonoccupational post-exposure prophylaxis, pre-exposure prophylaxis

## Abstract

Pre-exposure prophylaxis (PrEP) and nonoccupational post-exposure prophylaxis (nPEP) were found to be effective HIV biomedical interventions. However, several barriers to acceptance of these interventions were discovered among populations at risk for HIV, and the Coronavirus Disease 2019 (COVID-19) pandemic may also exacerbate these. The current scoping review aims to update information in regards to facilitators and barriers for PrEP and nPEP acceptability among key populations collected in the past two years and to identify any existing knowledge gaps during the time of the COVID-19 pandemic. Of 1453 studies retrieved, 16 met the final inclusion criteria. The review synthesized a range of individual, PrEP-specific, psychosocial, and health system factors that may affect the acceptability of PrEP or nPEP. The conclusion from this scoping review is that more research is needed to enable a comprehensive understanding of the determinants of acceptability of PrEP and nPEP in the context of COVID-19, particularly among PWID and FSWs.

## 1. Introduction

Post-exposure prophylaxis (PEP) with antiretroviral medications following a possible HIV infection is regarded as a preventative strategy. It must be taken within 72 h after exposure to HIV, once or twice daily for 28 days [1]. Since 1988, a few hospitals have started to provide zidovudine (ZDV) for post-exposure prophylaxis (PEP) to health care workers after occupational exposure to HIV. After the relevant safety and efficacy assessments, recommendations for PEP after occupational exposure to HIV have been adopted in many countries [2]. In addition, some countries also released guidelines recommending PEP for non-occupational exposure to HIV, such as having sex without condoms and sharing needles with people who inject drugs, this was termed nonoccupational post-exposure prophylaxis (nPEP) [3].

According to the definition by the World Health Organization (WHO), “Pre-exposure prophylaxis (PrEP) is the use of an antiretroviral drug to block the acquisition of HIV infection by uninfected people” [4]. The first clinical trials on PrEP were conducted in 2005, which focused on the effectiveness of PrEP among people who inject drugs, HIV sero-discordant couples, heterosexual men and women, women at higher risk of HIV exposure, and men and transgender women who have sex with men (MSM-TG) [5]. After reviewing the effectiveness and safety of PrEP from clinical trial data, WHO published guidance on PrEP administration in 2012, in which PrEP is recommended to key populations [6].

Pre-exposure prophylaxis (PrEP) and non-occupational exposure prophylaxis (nPEP) were effective HIV biomedical interventions. Nevertheless, several barriers to acceptance of these HIV biomedical interventions were found among populations at risk for HIV, such as female sex workers (FSW), people who inject drugs (PWID), and men who have sex with men (MSM) [7,8,9]. The use of PrEP for HIV prevention remains much below target, in 2020, only 28% of the target of three million in low- and middle-income countries was reached, achieving only 8% of the revised global 2025 target [10]. The barriers to the use of these HIV prevention strategies may have been exacerbated during the COVID-19 pandemic.

Up to the present (May 2022), more than 500 million people have been infected and almost six million died of Severe Acute Respiratory Syndrome Coronavirus 2 (SARS-CoV-2) worldwide [11]. The Coronavirus Disease 2019 (COVID-19) epidemic was declared a global pandemic by the World Health Organization (WHO) in 2020 [12]. Many public health efforts have been implemented to prevent the spread of coronavirus, such as lockdowns, staying at home orders, keeping social distance, and wearing a mask, et al. [13]. Globally, efforts to prevent the spread of COVID-19 have halted the growth of new cases, but have also led to unintended effects such as health care ramifications. The disruption of health care delivery caused by COVID-19 may have negative consequences for people’s health in addition to those caused by COVID-19 [14].

COVID-19 presents an HIV prevention challenge. A study in the United States indicated that nearly 82% of HIV clinics were either partially or fully closed during the COVID-19 pandemic [15]. In New York City, practically all in-person ambulatory visits were stopped in late March 2020. Outpatient primary healthcare clinics have fallen from roughly 2000 face-to-face visits per day to less than 100 by May 2020. Additionally, it was noted that providing adequate clinical care to PrEP-treated patients was jeopardized [16]. The World Health Organization and UNAIDS predicted that a disruption in condom supplies and peer education would leave people more vulnerable to a rise in HIV incidence [14].

Given that the impacts of COVID-19 on the health system related to HIV prevention and COVID-19 prevention measures evolved rapidly, we conducted a scoping review to update information with regards to facilitators and barriers of PrEP and nPEP acceptability among key populations collected in the past two years and identified any existing knowledge gaps during the COVID-19 pandemic. This could add to previous reviews in regards to the understanding of PrEP and nPEP acceptability and their correlates.

## 2. Methods

The PRISMA extension for scoping reviews was followed in writing this review [17].

### 2.1. Eligibility Criteria

To be included in the review, papers need to measure or focus on specific dimensions of acceptability of PrEP or nPEP. Peer-reviewed journal papers were included if they had the following: the end time of the data collection was after 1 January 2020, written in English, describing the influencing factors of oral daily use of PrEP or nPEP acceptability, studies assessing the actual provision and utilization of PrEP or nPEP were also included. Quantitative and mixed-method studies were included. Papers were not included if they were reviews, did not have full text, or did not meet the research aims.

### 2.2. Information Sources and Search Strategy

To identify potentially relevant documents, the following bibliographic databases were searched from 1 January 2020, to 13 April 2022: PubMed and Scopus. Combinations of the following search terms were used across all databases: PrEP, Pre-exposure prophylaxis, nPEP, post-exposure prophylaxis, non-occupational prophylaxis, PEP, HIV, human immunodeficiency virus, HIV, AIDS, acquired immunodeficiency syndrome, female sex workers, men who have sex with men, and people who inject drugs. The final search strategy for PubMed and Scopus can be found in the additional file (see Supplementary File). The final search results were exported into Zotero, and duplicates were removed.

### 2.3. Selection of Sources of Evidence

The selection of publications included in the study analysis was conducted in three stages. First, duplicate search results were removed from the Zotero file that contained database search results, and the remaining articles were compiled into a spreadsheet for the next stage of the review. Second, unpublished abstracts, dissertations, editorials, commentaries, and reviews were removed from the spreadsheet. Third, we limited the search results to studies focused on the acceptability of PrEP or nPEP through title and abstract reviews of the citations in the spreadsheet. Published quantitative or mixed-method research studies that met the above inclusion criteria underwent a full-text review.

### 2.4. Data Charting Process

To extract the variables, two reviewers jointly developed a data-charting form based on the PRISMA guidelines. Data were charted by two reviewers. Two reviewers iteratively discussed the results and adjusted the data-charting form.

### 2.5. Data Items

We extracted data on article characteristics, population, data collection year, acceptability measurement, barriers, and facilitators to acceptability. Based on previous studies, acceptability has been “conceptualized largely as a favorable ‘attitude’ towards a product, predisposing a person to be willing to take or use it” [18,19]. However, there is no consensus to measure acceptability. We stated the questions of assessing acceptability to reflect acceptability measurement. Table 1 summarizes the data from each study.

### 2.6. Synthesis of Results

We grouped the studies by type of influencing factors they reported, and summarized them into domains of individual, PrEP-specific, psychosocial, and health system factors.

## 3. Results

**Figure 1:**
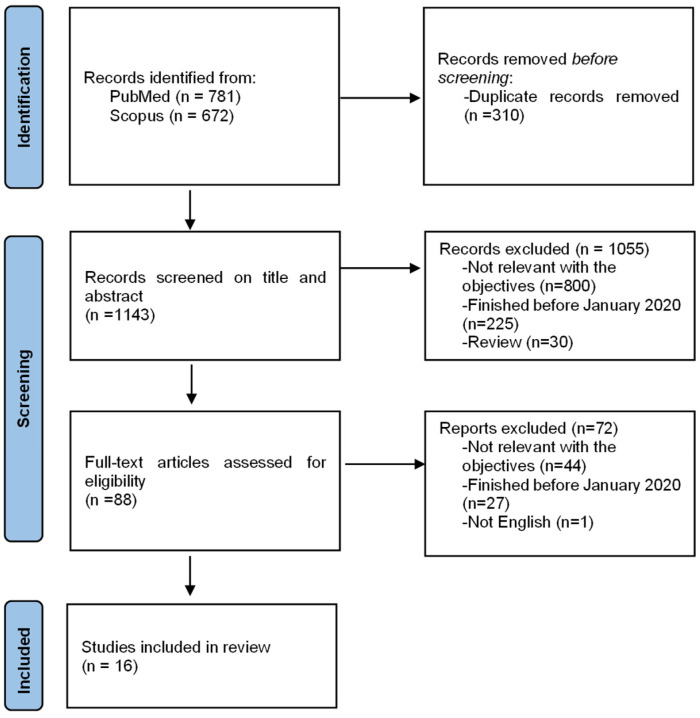
Flow diagram of study selection.

Sixteen studies met the inclusion criteria and were included in the final review (Figure 1). Most of the studies focused on MSM (n = 13), with three studies focusing on FSW and none on PWID. Most studies assessed the acceptability of PrEP (n = 15). One study assessed the acceptability of both PrEP and nPEP, and one only for nPEP. Of the outcomes of interest to this review, eight studies reported actual PrEP or nPEP use, one assessed actual and theoretical use, while the others reported theoretical use. These factors, which could potentially prevent or facilitate participants’ willingness to use or use PrEP or nPEP, fall into four different categories: within the individual, PrEP-specific factors, psychosocial, and health system domains.

### 3.1. Individual Factors

#### 3.1.1. Demographic and Socio-Economic Characteristics of Participants

Six studies discovered a relationship between PrEP acceptability and a variety of demographic characteristics. One study conducted in China showed that MSM whose marital status was separated/divorced/widowed and whose occupations were migrant worker/farmer, and government employees were more likely to use PrEP [24]. Age was an inconsistent factor across studies; young age was found to be a facilitator of PrEP acceptability in two studies among MSM and FSW, respectively [21,33]. However, according to another study conducted in Brazil, 18–24-year-old MSM were less likely to use PrEP [23]. A study conducted in four American cities showed Asian participants were less likely to use PrEP [31], while being born in Australia was a facilitator of acceptability [21], and Black respondents were less likely to use PrEP [23]. A study conducted in Ghana found different regions showed varied acceptability, and religion influenced acceptability as well [33]. Fewer years engaged in sex work had a positive effect on the acceptability of PrEP among female sex workers [32]. Socioeconomics was another domain to be concerned about. Believing PrEP is affordable was reported as one of the facilitators in a study [21].

The only two studies assessing nPEP showed that MSM who were married or cohabiting, had an HIV-positive sexual partner, and residing in Shenzhen were barriers. However, having an ‘HIV status unknown’ sexual partner and multiple sexual partners (≥2) were facilitators [24,35].

#### 3.1.2. Awareness and Knowledge

Participants’ knowledge of PrEP and HIV had a positive effect on the acceptability of PrEP. Three studies reported that having a higher level of PrEP knowledge or having heard of PrEP before increased the likelihood of accepting PrEP [20,30,33]. A study conducted in California, USA, showed MSM who were unaware of PrEP were less likely to use PrEP [28]. One study showed increasing HIV knowledge scores were associated with higher acceptability of PrEP [23]. One study showed being interested in initiating PrEP was positively associated with nPEP uptake [35].

#### 3.1.3. Behavioral Factors

Some of the behaviors related to HIV were also reported. Having been tested for HIV before was a facilitator for the acceptability of both PrEP and nPEP [24]. Another study, conducted in Ghana, showed that FSWs who were screened for STIs and had anal sex were more likely to use PrEP [33]. A study conducted in Shenzhen, China, showed that having sexual intercourse with women, preferring to find sexual partners in MSM venues, using Viagra, and receiving HIV-related services in clinics or MSM venues were all positively associated with nPEP uptake, whereas condom use in anal sex, lubricant use in annal sex, and rush popper use were all negatively associated with nPEP uptake [35].

### 3.2. PrEP-Specific Factors

Not being worried about side effects and believing PrEP was easy to remember to take were facilitators of acceptability among gay and bisexual men in Australia [21]. Other PrEP-specific factors were perceiving PrEP as an important prevention tool and believing that PrEP users took good care of themselves and others [20]. Furthermore, the more positive PrEP attitudes were the more this factor was related to a higher acceptability of PrEP [30]. On the contrary, a greater degree of PrEP stigma was negatively associated with the willingness to use PrEP [30].

### 3.3. Psychosocial Factors

Five studies reported psychosocial factors associated with the acceptability of PrEP. Higher social support was a facilitator among MSM in China and women engaged in sex work in southwest Uganda [24,32]. A study conducted among young MSM showed that higher scores in quality of life domains, such as physical health, psychological health, and environment; indicated higher odds of PrEP use [25]. Another study also showed that more descriptive norms and a greater degree of subjective norms were facilitators of willingness to use PrEP [30]. In one study conducted in China, a positive association between social support and the acceptability of nPEP was observed [24]. Higher internalized homonegativity levels were reported as the barrier factor in connection to PrEP acceptability [23].

### 3.4. Health System Factors

Residing in a PrEP desert (ZIP Codes with a one-way drive time of more than 30 min to the nearest PrEP-providing clinic) meant a lesser likelihood of PrEP usage [22]. Those who had community-based PrEP service delivery and motivational interviewing interventions were more likely to use PrEP [29,34]. Two studies reported COVID-19-related factors. One UK study showed that pandemic-related control measures were barriers to PrEP use [26]. However, another study showed that participants having physical reactions related to COVID-19 and reporting being close to people diagnosed with COVID-19 were more likely to use PrEP [27].

## 4. Discussion

This scoping review aimed at examining the determinants of acceptability of PrEP and nPEP among FSWs, PWID, and MSM in the past two years and identifying any existing knowledge gaps during the COVID-19 pandemic. In the review, we identified a limited number of published studies (n = 16) that met our objectives. Fifteen studies assessed PrEP, only two studies assessed nPEP, and one study contributed to both methods. The review synthesized a range of individual, PrEP- and nPEP-specific, psychosocial, and health system factors that might impact the acceptability of PrEP or nPEP.

More than 80% of the identified studies were conducted among MSM, three studies focused on FSWs, none on PWID. This may be because MSM suffered a high HIV incidence. In 2015, a study in the USA showed HIV prevalence among MSM was six times higher than that among PWID and more than 90 times higher than among heterosexuals [36]. However, considering the population size and the interaction with the general population, PWID and FSWs are still critical in regard to the spread of HIV. More research with intentional inclusion of those groups in PrEP and nPEP studies was required.

We found recent research assessing nPEP acceptability was lacking. The possible explanation is that nPEP has a longer history than PrEP, making evidence of its use and acceptability fairly saturated before January 2020. In 2005, the Center for disease control and prevention published the first nPEP guidelines [37]. Seven years later, in 2012, the WHO published PrEP guidelines [6]. However, in some developing areas, nPEP is still new, and research corresponding to the local situation is thus needed.

The findings of our review suggested that individual factors determined the acceptability of PrEP and nPEP. Demographic characteristics information can be used to identify a key population that needs help to improve the acceptability of PrEP or nPEP, such as Asian, black MSM in certain areas, or in regards to low-income groups. Increasing the awareness of PrEP and HIV knowledge may contribute to increasing the acceptability of PrEP. This indicates health providers should develop dissemination strategies to provide accurate education on PrEP and HIV. Some HIV-related behaviors such as rush popper use serves as barriers to nPEP use, thus nPEP information should be combined with recreational drug use reduction counselling.

Due to the small sample size of the studies assessing PrEP- and nPEP-specific factors, more research is needed in order to clarify the effects of those factors in connection to the acceptability of PrEP and nPEP. Social support was a facilitator toward the acceptability of PrEP and nPEP, and this finding is consistent with a previous systematic review conducted in 2016 [38]. This indicated that when conducting PrEP and nPEP programs there is a need to think about how partners and peers can be used to help the target population to accept PrEP and nPEP.

Some health system factors were identified in the reviewed studies. Enhancing the accessibility to prevention services may increase acceptability. However, the pandemic made it a challenge. Further studies are needed, for example exploring the application of mHealth in the provision of PrEP and nPEP services. COVID-19-related control measures reduced PrEP use; however, the fear of COVID-19 infection increased the likelihood to use PrEP. Due to the small sample size, it is hard to synthesize the impact of the COVID-19 pandemic on the acceptability of those preventions. Deeper research is needed.

All facilitator and barrier themes that we summarized in this scoping review, such as individual factors, psychosocial factors, and health system factors; were consistent with previous reviews. A recent review that looked at black men who have sex with men showed that key barriers to the PrEP care continuum included cost, HIV-related stigma, and side effects; whereas the facilitators included gaining PrEP awareness from social and sexual networks [39]. Another review revealed that individual, social, and health system domains were related to potential PrEP use [38]. However, we found that more psychosocial factors have been reported than before while earlier reviews found that the most reported barriers to use were safety, side effects, cost, and effectiveness [40,41]. Higher social support and higher scores in the quality of life domain facilitated PrEP acceptability, while higher internalized homonegativity levels diminished the use of PrEP. This indicates there is a need to explore whether people are more susceptible to psychosocial factors under the influence of the COVID-19 pandemic. In addition, our current review identifies PrEP- and nPEP-specific factors on the acceptability of PrEP and nPEP, and we also included COVID-19-related factors, which may add more information to the previous literature. Finally, through our review, we found that there were gaps in knowledge on studies related to PrEP and nPEP acceptability. Studies conducted among the other HIV high-risk populations, such as FSW and PWID, were limited. Furthermore, new studies relating to nPEP use are lacking, and studies assessing the impact of COVID-19 on potential PrEP and nPEP usage are scarce.

Several limitations were found in our study. There is no consensus on the measurement of acceptability, and this study includes studies that assessed the theoretical and actual use of PrEP and nPEP. Thus, findings may vary in practice. In addition, we recruited studies published after January 2020, which was the time of the COVID-19 outbreak. However, the timing of the COVID-19 outbreak was different in each region, and some peer-reviewed articles may have been missed. We only focus on the papers published during the COVID-19 period, the comparison with pre-pandemic studies was limited; therefore, caution should be used when explaining the results. Finally, we did not include qualitative studies, and this may have resulted in missing some relevant information. Nevertheless, this scoping review provides a synthesis of the findings of the determinants of PrEP and nPEP and identifies several existing knowledge gaps during a pandemic period, which may provide information for future study for health providers and policymakers to guide them in their formulating of decisions in regards to improving the acceptability of both prevention strategies in the context of COVID-19.

## 5. Conclusions

Individual, PrEP- and nPEP-specific, psychosocial, and health system factors may have an impact on the acceptability of PrEP and nPEP. However, our scoping review shows a dearth of evidence that can enable a comprehensive understanding of the determinants of acceptability of PrEP and nPEP in the context of COVID-19, particularly between PWID and FSW. In addition, acceptability toward PrEP and nPEP is expected to evolve with time. It is crucial to conduct more research observing determinants among the key populations for HIV in order to more effectively address obstacles to PrEP and nPEP acceptability.

## Figures and Tables

**Table 1:** Characteristics of included studies (ordered by year).

First Authors, Year	Location, Study Population, N	Collection Year	Acceptability Measurements	PrEP/nPEP	
				Barriers	Facilitators
1. PrEP ^1^ studies 1.1 MSM ^2^					
Hulstein et al. [20] (2022)	The Netherlands. Young MSM aged ≤25 years(n = 93),	2020	Are you planning to use PrEP in the coming 6 months?		Perceiving PrEP as an important prevention tool, a high level of PrEP knowledge, believing that PrEP users take good care of themselves and others
Chan et al. [21] (2022)	New South Wales. Australia. Gay and Bisexual Men (n = 1477)	2019–2020	“New forms of PrEP are currently under development. If all of these options were available, equally effective in preventing HIV, and had a similar cost, which of these would you want to use?”.		Younger age, being born in Australia, currently taking oral PrEP, not being worried about side effects, believing PrEP is affordable, believing that it is easy to remember to take pills, and not having difficulties with waiting for a PrEP appointment
Sharpe et al. [22] (2022)	The non-urban USA. MSM (n = 4792),	2020	In the past 12 months, have you taken PrEP?	Residing in PrEP desert (Structure barrier)	
Blair et al. [23] (2022)	Brazil. MSM (n = 2398),	2020	Are you taking or have you taken PrEP?	Increasing Homosexuality Scale scores, 18–24 yeas-old, Black respondents	Increasing HIV knowledge scores
Zhou et al. [24] (2022)	Guilin, China. MSM (n = 219)	2020–2021	Overall, how likely would you use PrEP?		Separated/Divorced/Widowed, Labourer, Migrant worker/Farmer, Government employee, Higher Social support, Tested HIV before
Liu et al. [25] (2022)	Nashville, Tennessee, and Buffalo, New York, USA. YMSM ^3^ (n = 347), convenience sampling	2019–2020	PrEP use (ever/current vs. never)		Quality of life domains: physical health, psychological health, and environment
Gillespie et al. [26] (2022)	Wales, UK. MSM (n = 60)	2019–2020	Actual PrEP use	The introduction of pandemic-related control measures	
Chen et al. [27] (2021)	Chicago, USA. Black MSM and transgender women (n = 222)	2020	Self-reported previous use of PrEP		Having physical reactions (eg, sweating and pounding heart) in regards to worries or problems related to COVID-19 and if they reported being in close proximity to a person who had been diagnosed with COVID-19.
Sevelius et al. [28] (2021)	California. USA. MSM (n = 185)	2017–2020	Actual PrEP use	Unaware of PrEP before enrolment	
Chan et al. [29] (2021)	US. MSM (n = 86)	2019–2020	PrEP uptake		Received motivational interviewing intervention
Mueses-Marín et al. [30] (2021)	Colombia. MSM (n = 287)	2020	If PrEP is effective in reducing the risk of HIV by 90%, and in the next 12 months PrEP was offered for free in Colombia, would you like to use PrEP to prevent HIV?	Greater degree of PrEP stigma	Higher PrEP knowledge, more positive PrEP attitudes, more descriptive norms, and greater degree of subjective norms.
Gordián-Arroyo et al. [31] (2020)	New York City, Birmingham, Chicago, and Seattle. USA. Adolescent MSM (n = 761), convenience sampling	2020	Would you take one pill a day to prevent HIV?” (0 = No, 1 = Yes)	Asian participants	
1. PrEP studies 1.2. FSW ^4^					
Witte et al. [32] (2022)	Southwestern Uganda. Women engaged in sex work (n = 283)	2019–2020	If PrEP were safe, effective, and free, how likely would you be willing to use it?		Fewer years engaged in sex work, greater perceived social support from family
Guure et al. [33] (2022)	Ghana. FSW (n = 5107). Time location sampling	2020	As PrEP has similar side effects to other drugs used to treat HIV, would you be willing to take it?	25 to 24 years (vs less than 25 years), Ahafo, Bono, Eastern, Greater Accra, Upper west region (VS Ashanti region)	Ever heard about PrEP, screened for STIs, Muslims and other religions (vs. Christians), and had anal sex.
Matambanadzo et al. [34] (2021)	Zimbabwean. Female sex workers (n = 19,407).	2020	Actual PrEP uptake		Community-based PrEP service delivery model is effective and can be adapted for long-term use.
2. nPEP ^5^ studies					
Zhou et al. [24] (2022)	Guilin, China. MSM (n = 219), RDS ^6^	2020–2021	Overall, how likely would you use nPEP?	Married or cohabited, Having an HIV-positive sexual partner	Higher Social support, Having HIV status unknown sexual partner, Tested HIV before
Wang et al. [35] (2022)	Shenzhen, China. MSM (n = 2833). time location sampling, RDS	2018–2020	Whether they have used PEP (Actual nPEP use)	Residence in Shenzhen, condom use in anal sex, lubricant use in anal sex, rush popper use	Having sexual intercourse with women, preferred finding sexual partners in MSM venues, multiple sexual partners (≥2), Viagra use, receiving HIV-related services in clinics or MSM venues, and interest in initiating PrEP.

^1^ PrEP: pre-exposure prophylaxis; ^2^ MSM: men who have sex with men; ^3^ YMSM: young men who have sex with men; ^4^ FSW: female sex worker; ^5^ nPEP: non-occupational post-exposure prophylaxis; ^6^ RDS: respondent-driven sampling.

## Data Availability

Not applicable.

## Supplementary Materials

The following are available online at https://www.mdpi.com/article/10.3390/ijerph191912605/s1, Supplementary File: Search Strategy.
