# Supplementary material for: Current Perspectives on the Determinants of Acceptability of Pre-Exposure Prophylaxis and Nonoccupational Post-Exposure Prophylaxis among People at Risk for HIV: A Scoping Review
Source: Int J Environ Res Public Health. 2022 Oct 2;19(19):12605. doi: 10.3390/ijerph191912605 (PMC9565962; doi:10.3390/ijerph191912605)
Supplement: Supplementary file 1 [file ijerph-19-12605-s001.zip › ijerph-1913614-supplementary.pdf]

Database	Literature Search Strategy
Scopus	(TITLE-ABS-KEY (prep) OR TITLE-ABS-KEY (pre-exposure AND prophylaxis) OR TITLE-ABS-KEY (iprex) OR TITLE-ABS-KEY (prep AND chemo-prophylaxis) OR TITLE-ABS-KEY (chemo-prophylaxis) OR TITLE-ABS-KEY (chemo-prevention) OR TITLE-ABS-KEY (npep) OR TITLE-ABS-KEY (post-exposure AND prophylaxis) OR TITLE-ABS-KEY (non-occupational AND post AND exposure AND prophylaxis) OR TITLE-ABS-KEY (pep) OR TITLE-ABS-KEY (truvada) OR TITLE-ABS-KEY (tenofovir) OR TITLE-ABS-KEY (emtricitabine))<br><br>AND<br><br>(ALL (hiv) OR ALL (human AND immunodeficiency AND virus) OR ALL (aids) OR ALL (acquired AND immunodeficiency AND syndrome)) AND (TITLE-ABS-KEY (willing*) OR TITLE-ABS-KEY (accep*) OR TITLE-ABS-KEY (perfer*) OR TITLE-ABS-KEY (use) OR TITLE-ABS-KEY (attitude))<br><br>AND NOT<br><br>(TITLE (review))<br><br>AND<br><br>(LIMIT-TO (PUBYEAR , 2022) OR LIMIT-TO (PUBYEAR , 2021) OR LIMIT-TO (PUBYEAR , 2020)
Pubmed	(((("PrEP"[Title/Abstract] OR "pre exposure prophylaxis"[Title/Abstract] OR "nPEP"[Title/Abstract] OR "post exposure prophylaxis"[Title/Abstract] OR "non occupational post exposure prophylaxis"[Title/Abstract] OR "PEP"[Title/Abstract]) AND 2018/01/01:3000/12/31[Date - Publication]<br><br>AND<br><br>("willing*"[Title/Abstract] OR "accep*"[Title/Abstract] OR "prefer*"[Title/Abstract] OR "attitude"[Title/Abstract] OR "use"[Title/Abstract])<br><br>AND<br><br>("hiv"[MeSH Terms] OR "hiv"[All Fields] OR ("hiv"[MeSH Terms] OR "hiv"[All Fields] OR ("human"[All Fields] AND "immunodeficiency"[All Fields] AND "virus"[All Fields]) OR "human immunodeficiency virus"[All Fields]) OR ("acquired immunodeficiency syndrome"[MeSH Terms] OR ("acquired"[All Fields] AND "immunodeficiency"[All Fields] AND "syndrome"[All Fields]) OR "acquired immunodeficiency syndrome"[All Fields] OR "aids"[All Fields]) OR ("acquired immunodeficiency syndrome"[MeSH Terms] OR ("acquired"[All Fields] AND "immunodeficiency"[All Fields] AND "syndrome"[All Fields]) OR "acquired immunodeficiency syndrome"[All Fields]))) NOT "review"[Title])<br><br>AND<br><br>((("female"[MeSH Terms] OR "female"[All Fields] OR ("female"[All Fields] AND "sex"[All Fields]) OR "female sex"[All Fields]) AND ("occupational groups"[MeSH Terms] OR ("occupational"[All Fields] AND "groups"[All Fields]) OR "occupational groups"[All Fields] OR "worker"[All Fields] OR "workers"[All Fields] OR "worker s"[All Fields])) OR "FSW"[All Fields] OR "csw"[All Fields] OR (("people s"[All Fields] OR "peopled"[All Fields] OR "peopling"[All Fields] OR "persons"[MeSH Terms] OR "persons"[All Fields] OR "people"[All Fields] OR "peoples"[All Fields]) AND "who"[All Fields] AND ("inject"[All Fields] OR "injectability"[All Fields] OR "injectant"[All Fields] OR "injectants"[All Fields] OR "injectate"[All Fields] OR "injectates"[All Fields] OR "injected"[All Fields] OR "injectible"[All Fields] OR "injectibles"[All Fields] OR "injecting"[All Fields] OR "injections"[MeSH Terms] OR "injections"[All Fields] OR "injectable"[All Fields] OR "injectables"[All Fields] OR "injection"[All Fields] OR "injects"[All Fields]) AND "drug"[All Fields]) OR (("inject"[All Fields] OR "injectability"[All Fields] OR "injectant"[All Fields] OR "injectants"[All Fields] OR "injectate"[All Fields] OR "injectates"[All Fields] OR "injected"[All Fields] OR "injectible"[All Fields] OR "injectibles"[All Fields] OR "injecting"[All Fields] OR "injections"[MeSH Terms] OR "injections"[All Fields] OR "injectable"[All Fields] OR "injectables"[All Fields] OR

	"injection"[All Fields] OR "injects"[All Fields]) AND ("drug users"[MeSH Terms] OR ("drug"[All Fields] AND "users"[All Fields]) OR "drug users"[All Fields] OR ("drug"[All Fields] AND "user"[All Fields]) OR "drug user"[All Fields])) OR "PWID"[All Fields] OR "IDU"[All Fields] OR ("sexual and gender minorities"[MeSH Terms] OR ("sexual"[All Fields] AND "gender"[All Fields] AND "minorities"[All Fields]) OR "sexual and gender minorities"[All Fields] OR ("men"[All Fields] AND "who"[All Fields] AND "sex"[All Fields] AND "men"[All Fields]) OR "men who have sex with men"[All Fields]) OR ("mens sana monogr"[Journal] OR "mater sociomed"[Journal] OR "msm"[All Fields]) OR ("sex workers"[MeSH Terms] OR ("sex"[All Fields] AND "workers"[All Fields]) OR "sex workers"[All Fields] OR ("sex"[All Fields] AND "worker"[All Fields]) OR "sex worker"[All Fields])) AND 2019/01/01:3000/12/31[Date - Publication]) AND (2020:2022[pdat])
